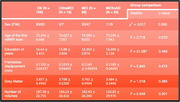# fMRI‐Complexity as a potential presymptomatic predictor of cognitive decline in Alzheimer's Disease

**DOI:** 10.1002/alz70856_098460

**Published:** 2025-12-24

**Authors:** Kay Jann, Ru Zhang, Jared Brown, Dilmini Wijesinghe, Kirsten M Lynch, Steven Cen, ADNI

**Affiliations:** ^1^ Mark and Mary Stevens Neuroimaging and Informatics Institute, University of Southern California, Los Angeles, CA, USA; ^2^ California State University, Fullerton, Fullerton, CA, USA; ^3^ Keck School of Medicine, University of Southern California, Los Angeles, CA, USA

## Abstract

**Background:**

Nonlinear statistical techniques, rooted in information theory, such as estimation of the complexity of fMRI timeseries using entropy methods are increasingly pertinent in the study of neurocognitive aging and neurodegenerative diseases. Changes in fMRI‐complexity are thought to indicate impairments in information processing capacity in brain areas [1] and in the progression of Alzheimer's Disease have been shown to decline with disease stage [2‐5]. Here we investigated the potential of fMRI‐complexity as an early marker of cognitive decline, with the goal of discerning participants with stable cognitive status from participants progressing in disease stage.

**Method:**

We identified subjects in ADNI that maintained normal cognition (NC), subjects with stable mild cognitive impairment (MCI), subjects converting from NC to MCI (NC2MCI), and subjects converting from MCI to Alzheimer's Disease (MCI2AD). Only fMRI from the initial visit were analyzed and preprocessed using the CONN‐toolbox. Complexity estimates were based on Multiscale Sample Entropy (MSE) in LOFT Complexity Toolbox [6], with four scales, pattern matching threshold m=2 and sensitivity threshold of r=0.3. Generalized linear model was used to compare across groups, adjusted for sex, age, education and motion. Dunnett's test performed pairwise comparisons with CN as the reference control.

**Result:**

There was no statistically significant difference in MSE for global grey‐matter. However, for four regions we observed a statistically significant effects across groups: middle frontal gyrus (MFG), superior frontal gyrus (SFG), lateral temporal lobe (LTL) and medial temporal lobe including hippocampus (MTL) with ANOVA *p*‐values of <0.001, <0.007, <0.004 and <0.05 respectively. Post‐hoc analysis revealed that in MFG the MCI2AD and CN2MCI had statistically significantly reduced MSE of ‐55.7 95% CI (‐91.08, ‐20.32) p:0.002 and ‐47.17 95% CI (‐86.55, ‐7.79) p:0.02 and in LTL only MCI2AD showed reduced MSE of ‐44.8 95% CI (‐80.97, ‐8.63) p:0.02.

**Conclusion:**

The converter groups showed significantly altered fMRI‐complexity while the stable groups showed no significant effect. These results demonstrate that fMRI‐complexity is sensitive to early presymptomatic changes in subjects that will transition from one stage to a more severe disease stage. This suggests that MSE could contribute to early diagnosis, prognostic monitoring and eventually precision care for Alzheimer's disease.